# Evaluation of ChatGPT-5 for CT Imaging in Canadian CT Head Rule-Positive Mild Traumatic Brain Injury: A Pilot Study

**DOI:** 10.3390/biomedicines14071555

**Published:** 2026-07-11

**Authors:** Marios Lampros, Eleni Romeo, Panagiota Zagorianakou, Spyridon Voulgaris, George A. Alexiou

**Affiliations:** 1 Department of Neurosurgery, University Hospital of Ioannina, 45100 Ioannina, Greece; marioslampros@gmail.com (M.L.); elenirome@gmail.com (E.R.); panzagorian@gmail.com (P.Z.); svoulgar@uoi.gr (S.V.); 2 Medical School, University of Ioannina, 45110 Ioannina, Greece

**Keywords:** mTBI, artificial intelligence, ChatGPT-5.1, large language models

## Abstract

**Background and Objectives:** Mild traumatic brain injury (mTBI) is a common cause of emergency department visits worldwide. Optimal decisions are essential for optimizing patient outcomes while minimizing unnecessary imaging. The present study explores the potential utility of ChatGPT-5.1 for predicting intracranial injury and determining CT necessity in mTBI. **Methods:** We evaluated adult patients with mild traumatic brain injury who met Canadian CT Head Rule (CCHR) criteria and underwent CT imaging during a two-year period. ChatGPT-5.1 was prompted under two frameworks: ER physician simulation and rule (literature)-based. Additionally, a probabilistic multi-variable logistic regression model was developed. ChatGPT-5.1’s performance was compared with that of human controls (consultants and residents) and the CCHR. Pairwise comparisons were conducted using McNemar’s test. **Results:** A total of 127 patients were included. ChatGPT-5.1’s recommendation for CT showed comparable performance to CCHR. For predicting CT findings, the probabilistic model achieved the highest performance (accuracy 74.0%), followed by consultants (accuracy 69.3%) and residents (66.9%). The rule (literature)-based model displayed the highest sensitivity (91.8%) but exhibited low specificity (22.2%), indicating a high rate of false positives despite its potential utility as a screening or decision-supporting tool. The ER physician simulation model demonstrated the lowest overall performance (accuracy 54.3%). Statistically significant differences were observed between the probabilistic model and both the ER physician and rule-based models (*p* < 0.05). Consultants outperformed the ER simulation model (*p* = 0.01), while their performance was similar to that of the probabilistic model (*p* = 0.44). **Conclusions:** ChatGPT-5.1 demonstrated performance comparable to the Canadian CT Head Rule in predicting the need for head CT imaging but showed lower accuracy in identifying intracranial injury. While the literature-based rule model achieved high sensitivity, its low specificity emphasizes that it acts primarily as a conservative screening aid rather than a definitive diagnostic tool in CCHR-positive mTBI patients.

## 1. Introduction

Mild traumatic brain injury (TBI) is a significant public health concern, as it accounts for the majority of TBI-related visits to the emergency department [1]. Effective triage of these patients, including the identification of cases requiring computed tomography (CT), is vital for optimal patient management. The decision to perform a head CT in patients with mTBI is currently based on clinical suspicion and clinical criteria. These criteria form the basis of the Canadian CT Head Rule (CCHR), which is used in conjunction with the physician’s clinical judgment in the emergency setting to determine whether a CT scan is necessary [2,3]. Despite their high sensitivity, these criteria have low to moderate positive predictive value, often leading to the overuse of CT scans, resulting in unnecessary radiation exposure and increased costs to the healthcare system [4].

Today, artificial intelligence (AI), particularly machine learning (ML) models, has been widely studied to predict prognosis and in-hospital mortality in patients with TBI, showing potential as a novel tool for evaluating these patients. However, most ML models require a large amount of patient data and specialized expertise to be effectively utilized [5]. Large Language Models (LLMs) are a type of AI model specifically designed to understand and generate human language. LLMs can answer questions, translate languages, summarize articles, generate images, and write code [6]. A type of LLM that has been widely used recently is the Chat Generative Pre-trained Transformer (ChatGPT) [7]. To date, the use of LLMs for the diagnosis and management of patients with TBI remains limited in the current literature [8,9,10,11].

In this pilot study, we evaluated patients who presented to the emergency department with CCHR-positive mTBI and assessed the potential use of ChatGPT-5.1 for determining the need for a head CT scan and its effectiveness in predicting intracranial trauma.

## 2. Materials and Methods

### 2.1. Study Design

In this study, we evaluated patients who presented to the emergency department of a tertiary hospital with mild traumatic brain injury (GCS 13–15) over a two-year period (2023–2025), during which neurosurgical consultation was requested. The ChatGPT-related analysis was performed using the GPT-5.1 model via the ChatGPT Plus subscription. No external plug-ins, apps, or third-party tools were enabled. The study was conducted in accordance with the Declaration of Helsinki and approved by the Institutional Review Board of the University Hospital of Ioannina (protocol no. 28/22-11-2023 for the prospective biomarker study; protocol no. 665/5-9-2025 for the retrospective use of data in AI-based analyses). All data were handled in compliance with applicable data protection regulations; datasets were fully anonymized, and all personal identifiers were removed prior to analysis.

Two outcomes were pre-specified:

1. Primary: Ability of ChatGPT-5.1 to predict CT-confirmed intracranial injury in CCHR-positive mTBI patients. Performance was evaluated across standard binary classification metrics (sensitivity, specificity, PPV, NPV, accuracy, and balanced accuracy).
2. Secondary: Concordance of ChatGPT-5.1’s head-CT recommendation with the CCHR within the same CCHR-positive population. Because CCHR-negative patients have a very high probability of a normal CT and do not require imaging, this comparison assessed whether ChatGPT-5.1 reproduces the conservative, high-sensitivity behavior of the CCHR within the decision-relevant subgroup, not whether it can independently select patients for imaging from an unselected mTBI population.

### 2.2. Patient Selection

We included adult (over 18) patients with mild TBI who presented in the emergency department within 12 h of the time of the accident. Patient selection followed a consecutive sampling approach, and data were prospectively collected as part of a broader institutional research protocol originally designed to investigate serum biomarkers in TBI. Only patients classified as CCHR-positive during the clinical evaluation and undergoing CT imaging were included to provide a reference standard for the detection of intracranial injury. The decision to restrict the cohort to CCHR-positive patients was deliberate and reflects the population of clinical interest. Patients who are CCHR-negative have an approximately 99% probability of a normal head CT and, by definition, do not warrant imaging. The diagnostic uncertainty, and therefore the clinically meaningful decision space, lies within the CCHR-positive population. It is precisely in this subgroup, in which the CCHR mandates imaging yet a substantial proportion of scans prove negative, that an adjunctive decision-support tool capable of further risk stratification would be most useful. ChatGPT-5.1 was therefore evaluated within this enriched, decision-relevant population rather than across an unselected mTBI cohort. The selection of this subgroup as the imaging reference standard aligns with landmark studies investigating the role of biomarkers in mTBI [11,12]. Therefore, all patients included in the present study had an indication for a CT scan according to CCHR criteria. The results of the CT scan were considered the gold standard for the comparisons performed in the present study. At our institution, CT scans are performed according to the CCHR, and the CT results (presence or absence of intracranial injury) were used as the gold standard against which ChatGPT-5.1 and CCHR predictions were evaluated. A head CT scan was considered positive when it demonstrated any intra-axial or extra-axial injury, including intracerebral contusion, epidural hematoma, subdural hematoma, traumatic subarachnoid hemorrhage, intraventricular hemorrhage, or any calvarial or skull base fracture.

### 2.3. Data Collection

From each patient, specific pre-defined demographic and clinical variables were recorded in an Excel document as a protocol of a study originally designed for evaluating the role of biomarkers in TBI. While the data were prospectively collected, the ChatGPT-5.1 analysis was performed retrospectively. Specifically, the patients’ age, sex, mechanism of injury, and trauma type (isolated or multi-trauma) were recorded. The mechanism of injury was classified as dangerous or non-dangerous based on the criteria defined by the Canadian CT Head rule [12]. Moreover, the dataset included clinical features, including GCS on arrival, loss of consciousness, amnesia, headache, vomiting episodes, post-traumatic seizure, any post-traumatic neurological deficit, presence of disorientation, alcohol/drug intoxication, and presence of scalp hematoma/laceration. Additionally, other factors recorded were the use of anticoagulants or antiplatelets, any coagulopathy disease, any cognitive impairment (e.g., dementia), and any prior history of neurosurgical intervention in the brain. Finally, the results of the head CT were recorded (presence or absence of intracranial injury).

### 2.4. Prompt Design and Predictive Model Development

ChatGPT-5.1 (OpenAI, San Francisco, CA, USA) was instructed: (1) To evaluate the need for a head CT scan in patients with mTBI and (2) to predict the presence or absence of intracranial trauma. Following data anonymization, standardized prompts were developed, incorporating input from all parameters recorded in the Excel documents. All parameters were encoded in a binary form except for continuous variables. Binary responses were also selected to allow direct comparability with the Canadian CT Head Rule and human raters and to facilitate statistical analysis. The prompt structure is presented in Supplementary Material S1.

### 2.5. CT Recommendation

ChatGPT-5.1 was instructed to simulate the decision of whether a cranial CT scan should be performed based on the patient’s clinical features. The exact prompt had the following structure: “Based on the clinical data provided, would you recommend performing a head CT scan to evaluate for possible intracranial injury in this patient presenting to the emergency department?”. The model was requested to respond in a binary format, with “1” indicating that a CT is recommended and “0” indicating that a CT scan is not recommended.

### 2.6. Prediction of Intracranial Injury

To evaluate the ability of ChatGPT-5.1 in predicting the presence or absence of intracranial trauma in patients with mTBI, the model was asked to respond with “1” indicating the presence of intracranial trauma, and “0” indicating the absence of intracranial trauma, based on two different clinical settings/frameworks:

1. ER physician simulation: In that mode, ChatGPT-5.1 simulated an ER physician’s clinical reasoning. For each case, the model received clinical data as input and was asked to make a decision based on general clinical reasoning patterns to inform its decision. The exact prompt had the following structure: “Based on the clinical data provided, and applying your clinical judgment as an emergency department physician, does this patient have a sustained traumatic intracranial injury?”.
2. Rule-based model: In that mode, ChatGPT-5.1 was instructed to predict the presence of intracranial trauma based on clinical-decision rules available in the literature. No specific set of rules was directly instructed by the investigators. Instead, the model was prompted to adopt its internal representation of clinical decision rules. That exact prompt had the following structure: “Based on the clinical data provided, and applying a rule-based approach and predefined clinical criteria, does this patient have a sustained traumatic intracranial injury?”.

The two frameworks were designed to be conceptually distinct. The ER physician simulation elicited holistic clinical reasoning, analogous to an individual clinician’s diagnostic gestalt, without reference to any explicit rule set. In contrast, the rule-based framework instructed the model to apply explicit, literature-derived decision criteria. This contrast was intended to isolate the contribution of rule application versus open-ended clinical judgment to the model’s predictions.

### 2.7. Logistic Regression Model

Probabilistic (Statistical) model: A conventional multivariate logistic regression model was generated to compare its efficacy with that of ChatGPT-5.1 (Supplementary Material S2).

### 2.8. Clinical Data Abstraction and Human Control

To evaluate ChatGPT-5.1’s predictive capability against that of human controls, two neurosurgery residents (PGY-3 and 4) and two attendings were blinded to the CT results and tasked with predicting the presence or absence of traumatic intracranial injury. Clinical data were originally collected prospectively from emergency department physicians as part of an institutional research protocol investigating serum biomarkers in traumatic brain injury and were recorded in a structured Excel database. For the present analysis, relevant clinical variables were retrospectively abstracted by two neurosurgery residents. Although CT results were included in the original dataset, they were intentionally withheld during abstraction and prompt generation to ensure blinding. The dataset was randomly divided and independently distributed to two neurosurgery residents and, separately, to two attending physicians (attendings). Each evaluator reviewed a random set of cases and recorded their decision individually, based solely on the structured clinical data, blinded to CT findings and the responses of other raters. CT outcomes were extracted separately from the original dataset at a later stage and used as the reference standard for diagnostic performance comparison. To minimize bias, the patients’ IDs were blinded.

### 2.9. Statistical Analysis

Continuous variables were expressed as mean ± standard deviation (SD) or median. Categorical variables were presented as frequencies and percentages. The differences between the categorical variables were evaluated using the chi-square test and Fisher’s exact test. A McNemar’s test was conducted to compare the classification outcomes of the different models on paired data. A *p*-value < 0.05 was considered statistically significant. Because all evaluated approaches (ChatGPT-5.1, the human raters, and the CCHR) produced binary (0/1) outputs rather than continuous probabilities or risk scores, we additionally report the balanced accuracy, defined as the arithmetic mean of sensitivity and specificity, for each model.

## 3. Results

The present cohort included 127 patients, comprising 82 males and 45 females. The mean age of the patients was 61.9 (±19.6) years. The majority of patients, 107 cases (84.3%), had a 15 GCS score, while 12 (9.4%) patients had a 14 GCS score, and the remaining 8 (6.3%) patients had a 13 GCS score. A summary of the patient’s demographic and clinical features is presented in Table 1.

### 3.1. CT Recommendation

By using the prompt for the CT recommendation, the model achieved 59.06% accuracy, 93.15% sensitivity, 59.13% positive predictive value (PPV), and 58.33% negative predictive value (NPV). The model’s performance was similar to that of the CCHR (McNemar’s test, *p* = 0.61). Similarly, the other diagnostic performances of CCHR were similar to the studied ChatGPT-5.1 model. Table 2 presents the diagnostic metrics of ChatGPT-5.1 and the CCHR. The balanced accuracy for the CT-recommendation task was comparable between the two approaches (ChatGPT-5.1, 53.1%; CCHR, 51.4%). It should be emphasized that, because every patient in the cohort was CCHR-positive by design, the diagnostic metrics reported for the CCHR in this task are constrained: the rule recommended imaging for essentially the entire cohort, so its apparent discrimination against the CT-confirmed outcome is limited, and these figures should be interpreted as descriptive rather than as an unbiased estimate of CCHR performance in an unselected mTBI population. Accordingly, the comparison should be read as an assessment of whether ChatGPT-5.1 can reproduce the conservative, high-sensitivity behavior of the CCHR within the decision-relevant CCHR-positive population, rather than as a head-to-head test of which tool better selects patients for imaging. The cohort was CCHR-positive by inclusion, as imaging was based on the treating physician’s real-time application of the rule at the bedside. For the present analysis, the CCHR was additionally re-derived post hoc from the structured variables recorded in the dataset. Because several CCHR criteria are examination-dependent and may be incompletely captured as discrete fields in a structured dataset, this re-derivation reclassified 2 of the 73 CT-positive patients as CCHR-negative, yielding a CCHR sensitivity of 97.3% rather than 100%. These cases reflect changes in the clinical picture shortly after the initial examination, where symptoms evolved during observation and were not reflected in the recorded data.

### 3.2. Prediction of Intracranial Injury

**Figure 1:**
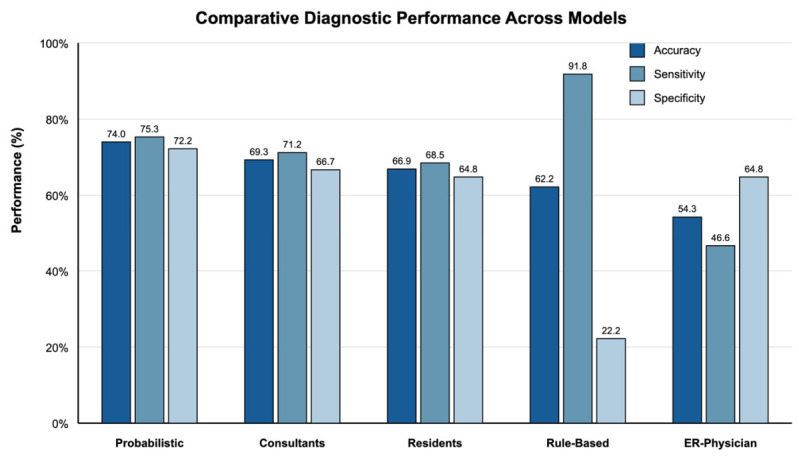
Comparative diagnostic performance of AI frameworks and human evaluators.

The probabilistic model demonstrated the highest accuracy (74.02%) with a sensitivity of 75.34% and specificity of 72.22%. The model also had a PPV of 78.57% and an NPV of 68.42%. The consultant’s prediction achieved an accuracy of 69.29%, with a sensitivity of 71.23% and a PPV of 74.29%. Residents’ predictions also exhibited similar sensitivity (68.49%) and PPV (72.46%). The ER physician simulation demonstrated lower performance (accuracy = 54.33%), with reduced sensitivity (46.58%) and NPV (47.30%). The rule-based model had the highest sensitivity (91.78%) but low specificity (22.22%). A summary of diagnostic performance metrics is presented in Table 3 and Figure 1.

### 3.3. Models Comparison

Pairwise comparisons of the different models showed that the Consultant’s and Resident’s predictions did not differ significantly (*p* = 0.77). Likewise, the comparison between Consultant and rule-based models (*p* = 0.26), Consultants and the probabilistic model (*p* = 0.44), Residents and rule-based models (*p* = 0.49), Residents and the probabilistic model (*p* = 0.22), and ER physician simulation versus the rule-based model (*p* = 0.30) also revealed no significant differences. In contrast, a statistically significant difference was noted between the Consultant’s predictions and the ER physician simulation model (*p* = 0.014), the ER physician simulation model and the probabilistic model (*p* = 0.002), and the rule-based model and the probabilistic model (*p* = 0.03). Finally, the comparison of the residents’ predictions versus the ER physician simulation model (*p* = 0.051) was close to being statistically significant. A summary of models’ comparison using the McNemar test is presented in Table 4.

### 3.4. Differentiating Factors

To identify clinical features that contributed to the differences between models, a pairwise disagreement variable was defined for each patient. For each input variable, a Chi-square test was applied using a 2 × 2 table to determine whether the distribution of that feature significantly differed between agreement and disagreement cases. Headache (*p* = 0.007), amnesia (*p* = 0.04), and disorientation (*p* = 0.03) were statistically significant factors associated with the observable difference between consultants and the ER physician simulation model, with all three factors being considered more important in the consultant group compared to the ER physician simulation model. Loss of consciousness (*p* = 0.0008), vomiting (*p* < 0.0001), and disorientation (*p* = 0.013) were significantly associated with model disagreement between the rule-based and probabilistic models, with these variables selected by the rule-based model but not chosen by the probabilistic model. Finally, no significant differentiation factor was observed between the ER physician simulation and the probabilistic model.

## 4. Discussion

To our knowledge, this is the first study to assess the efficacy of ChatGPT-5.1 as a decision support tool for patients with mTBI. Overall, ChatGPT-5.1 demonstrated similar efficacy to the Canadian head CT rule in determining the need for a head CT scan. ChatGPT-5.1 generally underperformed compared to human evaluators in predicting intracranial trauma. Still, the rule-based model demonstrated the highest sensitivity from both human evaluators and other models, making it a potentially useful decision-supporting tool.

The CCHR is a widely utilized clinical decision rule for assessing the necessity of a CT scan in patients with mTBI [12]. Its high sensitivity makes it suitable for ruling out the presence of intracranial trauma. However, because the CCHR was initially designed to optimize sensitivity and exclude intracranial trauma, it sacrifices specificity and diagnostic precision [13]. Consequently, the model often leads to high rates of unnecessary imaging. In previously conducted large-scale cohorts, the specificity of the CCHR has ranged from 50% to 70%, while the PPV has been reported to be approximately 10%, indicating that most of the CT scans recommended by the CCHR are negative for intracranial trauma [14,15]. Over the past few years, with the widespread use of machine learning tools, several efforts have been made to develop accurate clinical decision models for patients with traumatic brain injury [16,17]. However, the development of these models often requires structured data inputs, extensive processing, and technical infrastructure, which may not be broadly available. In contrast, LLMs such as ChatGPT-5.1, despite their ability to process clinical data in natural language form and generate outputs, have only been preliminarily studied in patients with mTBI. In the present study, ChatGPT-5.1, with the standard, physician-friendly prompts, demonstrated comparable diagnostic performance to the CCHR when asked to determine the need for a CT scan. To determine the reasons behind ChatGPT-5.1’s responses, it was introduced to explain the basis for its outputs. ChatGPT-5.1 stated that its responses were based on established clinical rules that exist in the literature, including CCHR [12], New Orleans Criteria, and National Institute for Health and Care Excellence (NICE) guidelines [18,19,20]. In this respect, the balanced accuracy of the CT-recommendation task (53.1% for ChatGPT-5.1) was comparable to that of the CCHR (51.4%) within this cohort. A balanced accuracy close to 50% for both approaches does not indicate diagnostic failure but rather reflects the intended behavior of a high-sensitivity safe-triage rule: because every patient was CCHR-positive by design and the CCHR flags virtually all such patients for imaging, neither approach can meaningfully discriminate CT-positive from CT-negative cases within this constrained population. Therefore, ChatGPT-5.1 reproduces the conservative triage behavior of the CCHR, which is designed to safely exclude intracranial injury rather than to serve as a diagnostic test. It is precisely for this reason that the discriminative ability of the model was subsequently evaluated using the distinct ER physician simulation, rule-based, and probabilistic prompting frameworks presented below.

In assessing ChatGPT’s performance in predicting the presence of intracranial trauma, the models displayed variable outcomes based on the prompt framework they were instructed to follow. The probabilistic-logistic regression model achieved the highest overall performance, followed by the rule-based and ER physician simulation models, which showed similar efficacy. The increased performance could be potentially attributed to the probabilistic model’s ability to statistically assess the contribution of each symptom to the outcome [16]. On the contrary, the rule-based model was likely based on a fixed threshold and demonstrated similar diagnostic performance to the CCHR. To further clarify this, ChatGPT was asked to explain the reasoning behind its answers in the rule-based model and confirmed that it was based on the CCHR, which was designed to exclude the presence of intracranial injury [12]. Finally, the ER physician model, which was based on general clinical reasoning and aimed to simulate an emergency physician’s actions rather than following predefined clinical rules, demonstrated the lowest performance. Interestingly, the ER physician model demonstrated lower performance than that of residents and consultants, with headache, amnesia, and disorientation being the variables related to the disagreement between the ER physician simulation model and consultants’ responses, as these factors were considered more important in the consultant group. The latter may suggest that without structured logic or defined thresholds, ChatGPT-5.1 fails to replicate the complex, experience-driven judgment of clinicians [21]. It is important to emphasize that, in the context of mTBI triage, overall accuracy is at best a secondary endpoint: a tool that misclassifies a substantial fraction of intracranial injuries as negative cannot be considered clinically usable, irrespective of its accuracy, because the cost of a missed injury is severe. Sensitivity is therefore the primary metric, and most of the evaluated approaches, with the exception of the rule-based model, yielded unacceptable amounts of sensitivity for improvements in specificity. Consequently, the main finding of the present study is the high sensitivity of the rule-based framework rather than the comparatively modest accuracy figures. We retained the ER physician simulation despite its weak performance because the contrast between unconstrained clinical reasoning and explicit rule application is itself informative: its underperformance, together with the divergence on variables such as headache, amnesia, and disorientation, helps to delineate the conditions under which a language model can or cannot approximate clinical reasoning, which is a relevant question for a pilot, hypothesis-generating evaluation.

The application of LLMs in medicine, particularly within emergency departments (EDs) for patients with TBI, has not been studied in depth. Yigit et al. evaluated ChatGPT as an adjunct tool to support clinical decision-making by applying it to 17 case scenarios that did not involve real patient data. They compared the model’s responses to decisions made by experienced clinicians and found that AI-based decision-making could be a novel tool in the management of TBI patients. However, they also observed that ChatGPT made critical errors, such as missing or delaying the recognition of major injuries, in approximately one-third of the patients [10]. In our study, ChatGPT-5.1 performance, when introduced to act as an ER doctor and under a rule-based framework, displayed lower efficacy when compared with consultants and neurosurgery residents. Similarly, in another recent study regarding the evaluation of ChatGPT-5.1 in predicting six-month outcomes following moderate and severe TBI, the authors observed that ChatGPT-5.1 responses performed lower in predicting primary and secondary outcomes compared to established rule-based models and specialized physicians [9]. However, ChatGPT-5.1 has been found to be a promising triage tool for patients in the emergency department. In a recent study involving 745 patients, ChatGPT was studied as a potential triage tool for patients visiting the ED for various reasons. Based on its responses, patients were categorized according to the Emergency Severity Index (ESI) and compared with categorizations made by experienced emergency physicians. The results showed that ChatGPT had higher accuracy in the more severe ESI categories, suggesting a potential role in patient triage [22]. Similar findings supporting the potential use of AI in patient triage in the emergency setting have been reported in the literature [23,24]. Hence, further studies should be performed on the spectrum of applications of ChatGPT-5.1 in mTBI, as it is possible that several currently unexplored factors, including prompt structure and clarity of the question, could influence the ChatGPT-5.1 performance. Potential strategies to improve ChatGPT performance may include the inclusion of additional clinical and laboratory findings, such as the use of TBI biomarkers (GFAP, S100, UCH-L1). Moreover, several studies have observed that routine, cost-effective laboratory parameters such as blood glucose and neutrophil to lymphocyte ratio (NLR) may assist in the triage of patients with TBI [25,26]. Finally, fine-tuning of ChatGPT, typically defined as the process of adapting an LLM to a specific clinical context by training it on targeted datasets of structured patient cases, may enhance the diagnostic performance of the model [27].

### Limitations

The present study has several limitations. First, although data collection was prospective, the analysis was conducted retrospectively at a single tertiary center, and the sample size remained relatively small. Moreover, there is potential sample bias, as it only included patients who underwent a CT scan and requested a neurosurgical consultation. Hence, the sample may represent a subgroup of mTBI with more severe symptoms. This design limited the ability to fully assess ChatGPT’s performance, and future studies should instead use clinically significant TBI as the more appropriate outcome measure. Another limitation is the use of binary outcomes and the absence of stratification by injury severity, as no separate analyses were performed according to individual GCS scores. Although this method allows for direct comparison between the different models, it may lead to simplified and non-graded outputs that do not fully reflect real clinical practice, where in cases of intermediate risk the final decision is often influenced by clinical judgment and experience. Future studies with larger cohorts should explore model performance in more complex decision-making scenarios rather than strictly dichotomized outcomes. A further limitation concerns the reproducibility of LLM outputs. Because these models are stochastic and are updated continuously by their developers, identical prompts may yield different responses across sessions or model versions, and we did not formally quantify the stability of ChatGPT-5.1’s answers through repeated prompting. This concern is inherent to the pilot nature of the present work and should be addressed prospectively in future studies through systematic test–retest evaluation and reporting of output concordance. Relatedly, our analyses did not account for the potential influence of prompt linguistics; subtle differences in wording, such as whether a query is framed using a verb or an adjectival construction, may affect model behavior in ways that have not yet been systematically studied and that we could not directly evaluate, as model updates over the study period would confound any retrospective comparison of alternative phrasings. In addition, the human control group consisted of neurosurgeons and neurosurgery residents practicing within a single department; clinicians who train and work together tend to share decision-making patterns and may therefore covary in their judgments, and predicting intracranial pathology from non-imaging data is intrinsically difficult, which is precisely the rationale underlying imaging decision rules such as the CCHR. This represents a genuine limitation of the comparison; however, consistent with the exploratory, single-center, pilot design of the study, it is reported here as a constraint on interpretation rather than a definitive benchmark, and future multicenter work should incorporate larger and more heterogeneous panels of independent evaluators.

## 5. Conclusions

In this pilot study, ChatGPT-5.1 reproduced the conservative, high-sensitivity behavior of the CCHR when asked whether a head CT was warranted. Because the cohort consisted exclusively of CCHR-positive patients, this observation should be regarded as hypothesis-generating rather than as evidence that the model can independently guide the decision to image. For the prediction of intracranial injury, the ER physician simulation and rule-based frameworks underperformed relative to consultants and residents in overall agreement. The rule-based model achieved the highest sensitivity among all evaluated approaches, albeit at the cost of low specificity, suggesting a possible role as a conservative screening adjunct rather than a standalone diagnostic tool. These preliminary findings warrant confirmation in larger, multicenter, prospective studies before any clinical application can be considered.

## Figures and Tables

**Table 1:** Baseline demographic and clinical features.

Parameters	Value (%)
Total Patients	127
Mean Age (±SD)	61.9 (±19.6)
Sex (Male)/(Female)	82 (64.6%)/45 (35.4%)
Isolated Trauma/Polytrauma	107 (84.3%)/20 (15.7%)
Dangerous/Non Dangerous Trauma Mechanism	43 (33.85%)/84 (66.15%)
Loss of Consciousness	39 (30.7%)
Amnesia	44 (34.6%)
Vomiting	27 (21.3%)
Headache	60 (47.2%)
Seizure	1 (0.8%)
Disorientation	21.0 (16.7%)
Alcohol/Drug Intoxication	13 (10.2%)
Scalp Hematoma/Laceration	32 (25.2%)/47 (37.0%)
Anticoagulants Use	8 (6.3%)
Antiplatelets Use	15 (11.8%)
History of Cognitive Impairment (Dementia)	9 (7.1%)
Positive CT Findings	73 (57.5%)
Negative CT Findings	54 (42.5%)

**Table 2:** Concordance of head CT recommendations between the Canadian CT Head Rule (clinical decision-guidance tool) and ChatGPT-5.1 in Canadian CT Head Rule-positive patients.

Performance Metric	Canadian CT Head Rule (95% CI)	ChatGPT-5.1 (95% CI)
Sensitivity	97.3% (90.5–99.7)	93.2% (85.4–97.4)
Specificity	5.6% (1.2–15.4)	13.0% (5.6–24.2)
PPV	58.2% (48.9–66.9)	59.1% (49.6–68.0)
NPV	60.0% (32.3–83.7)	58.3% (36.6–77.9)
Accuracy	57.5% (48.4–66.2)	59.0% (50.0–67.8)
Balanced Accuracy	51.4%	53.0%

PPV: positive predictive value, NPV: negative predictive value. Note: While the CCHR determination for study inclusion was based on bedside evaluation, the metrics presented here were re-derived using the CCHR algorithm within the structured database. As the cohort was CCHR-positive by design, these results are constrained and serve as a descriptive analysis rather than an unbiased evaluation of CCHR performance in a general population.

**Table 3:** Comparison of predictive models for intracranial injury detection in mild head trauma.

Diagnostic Metric	Consultants (95% CI)	Residents (95% CI)	ER Physician Simulation (95% CI)	Rule-Based Model (95% CI)	Probabilistic Model (95% CI)
Accuracy	69.3% (60.5–77.2)	66.9% (58.0–75.1)	54.3% (45.2–63.2)	62.2% (53.3–70.5)	74.0% (65.5–81.3)
Sensitivity	71.2% (59.3–81.2)	68.5% (56.4–78.9)	46.6% (34.7–58.8)	91.8% (82.7–96.9)	75.3% (63.8–84.8)
Specificity	66.7% (52.9–78.6)	64.8% (50.9–77.0)	64.8% (50.9–77.0)	22.2% (12.0–36.0)	72.2% (58.4–83.5)
PPV	74.3% (62.9–83.6)	72.5% (60.4–82.5)	64.1% (49.8–76.9)	61.5% (51.0–71.2)	78.6% (66.4–87.5)
NPV	63.2% (49.3–75.6)	60.3% (46.4–73.0)	47.3% (34.1–60.8)	66.7% (44.7–84.4)	68.4% (54.8–80.1)
Balanced Accuracy	68.95%	66.65%	55.70%	57.00%	73.78%

PPV: positive predictive value, NPV: negative predictive value.

**Table 4:** Agreement analysis between clinical and AI-based predictions using the McNemar test.

Comparison	McNemar *p*-Value
Consultants’ vs. Residents’ predictions	0.77
Consultants’ predictions vs. ER physician simulation model	0.01
Consultants’ prediction vs. Rule-based model	0.26
Consultants’ predictions vs. Probabilistic model	0.44
Residents’ predictions vs. ER physician simulation	0.05
Residents’ predictions vs. Rule-based model	0.49
Residents’ predictions vs. Probabilistic model	0.22
ER physician simulation vs. Rule-based model	0.30
ER physician simulation vs. Probabilistic model	0.002
Rule-based vs. Probabilistic model	0.035

## Data Availability

The data presented in this study are available on request from the corresponding author.

## Supplementary Materials

The following supporting information can be downloaded at: https://www.mdpi.com/article/10.3390/biomedicines14071555/s1, Supplementary Materials S1: Prompt structure used for ChatGPT-5.1 queries, including the CT recommendation prompt, the ER physician simulation prompt, and the rule-based prediction prompt; Supplementary Materials S2: Logistic regression model details, including variable selection procedure, final model coefficients with 95% confidence intervals, and model statistics.
