# Supplementary material for: Evaluation of ChatGPT-5 for CT Imaging in Canadian CT Head Rule-Positive Mild Traumatic Brain Injury: A Pilot Study
Source: Biomedicines. 2026 Jul 11;14(7):1555. doi: 10.3390/biomedicines14071555 (PMC13407308; doi:10.3390/biomedicines14071555)
Supplement: Supplementary file 1 [file biomedicines-14-01555-s001.zip › biomedicines-4344414-supplementary.pdf]

Supplementary Material S1

Template:

Patient Information

Age: [ ], Sex: [ ], Mechanism of injury: [dangerous / non-dangerous], Trauma type: [isolated / multi-trauma], GCS on arrival: [ ], Loss of consciousness: [yes/no], Amnesia: [yes/no], Headache: [yes/no], Vomiting episodes: [ ], Post-traumatic seizure: [yes/no], Post-traumatic neurological deficit: [yes/no], Disorientation: [yes/no], Alcohol or drug intoxication: [yes/no], Scalp hematoma or laceration: [yes/no], Anticoagulant/antiplatelet use: [yes/no], Coagulopathy disease: [yes/no], Cognitive impairment: [ ], Prior neurosurgical intervention: [yes/no].

Outputs (All responses must be provided strictly in binary format, where 1 = Yes and 0 = No)

1. **CT Recommendation (Triage Decision):**
   Based on the clinical data provided, would you recommend performing a head CT scan to evaluate for possible intracranial injury in this patient presenting to the emergency department?
   Response: [ ]
2. **Intracranial Injury Prediction (ER Physician Simulation):**
   Based on the clinical data provided, and applying your clinical judgment as an emergency department physician, does this patient have a sustained traumatic intracranial injury?
   Response: [ ]
3. **Intracranial Injury Prediction (Rule-Based Model):**
   Based on the clinical data provided, and applying a rule-based approach and predefined clinical criteria, does this patient have sustained traumatic intracranial injury?
   Response: [ ]

Examples of Responses

Example 1.

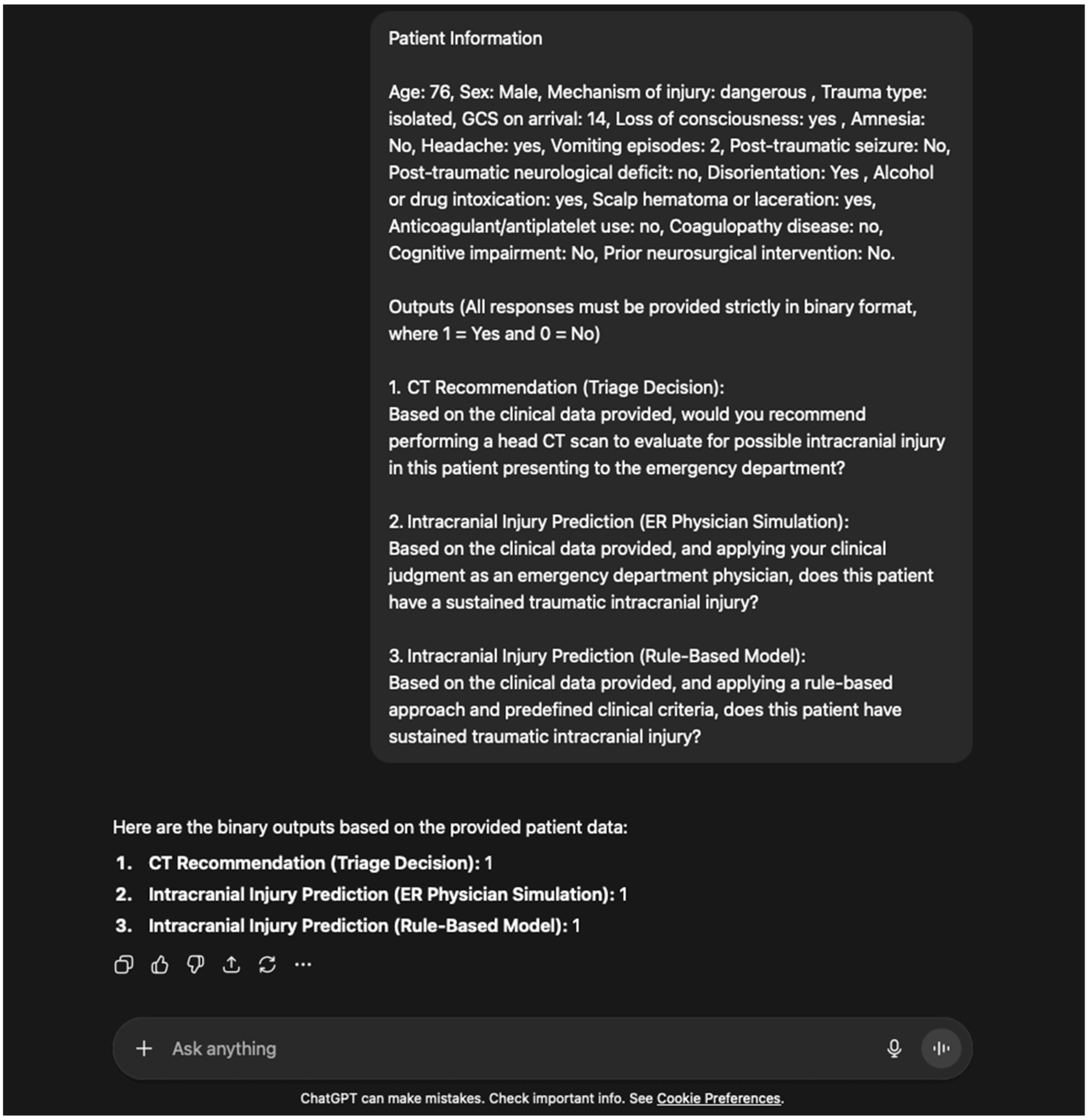

Example 2

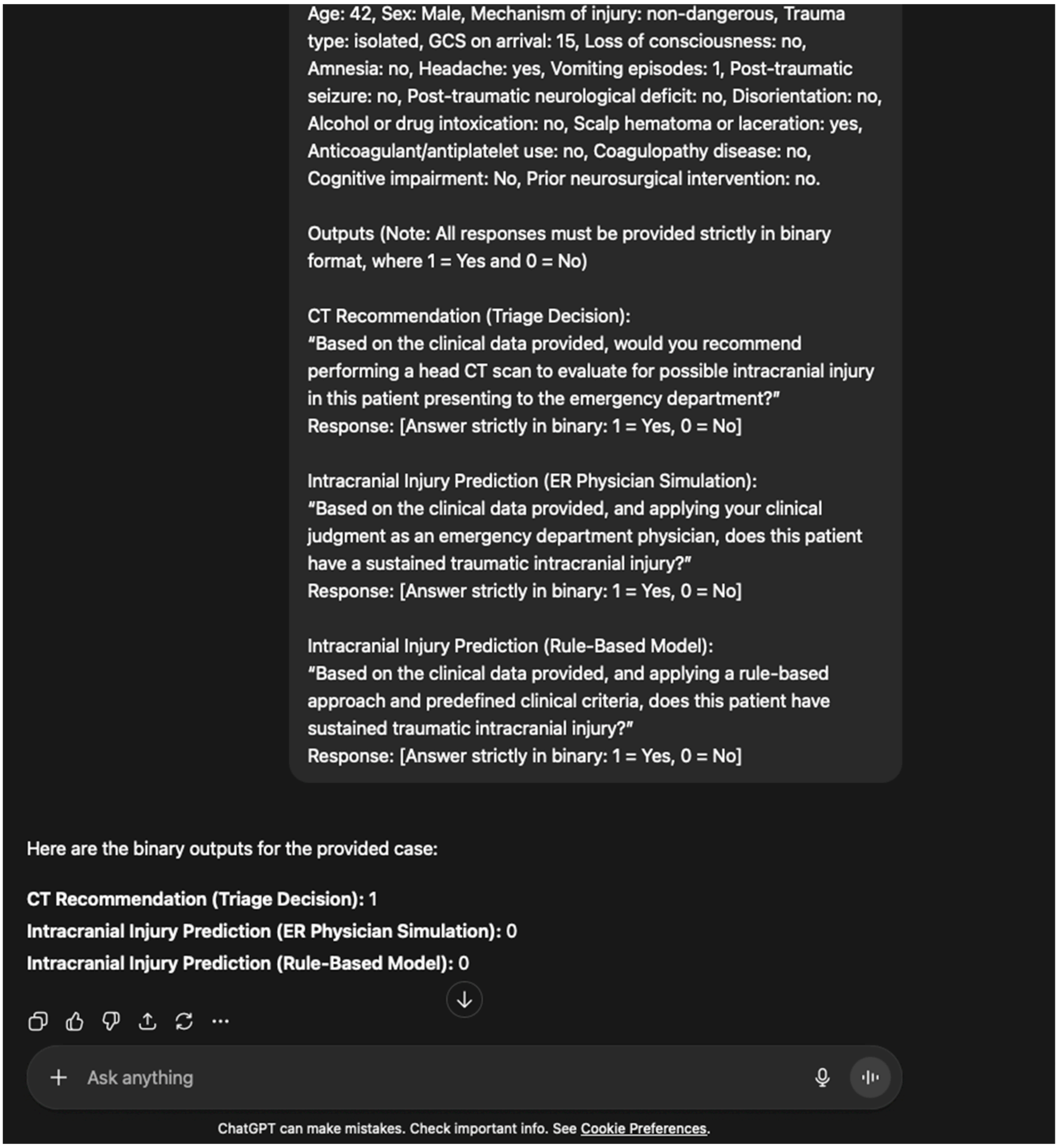
Age: 42, Sex: Male, Mechanism of injury: non-dangerous, Trauma type: isolated, GCS on arrival: 15, Loss of consciousness: no, Amnesia: no, Headache: yes, Vomiting episodes: 1, Post-traumatic seizure: no, Post-traumatic neurological deficit: no, Disorientation: no, Alcohol or drug intoxication: no, Scalp hematoma or laceration: yes, Anticoagulant/antiplatelet use: no, Coagulopathy disease: no, Cognitive impairment: No, Prior neurosurgical intervention: no.

Outputs (Note: All responses must be provided strictly in binary format, where 1 = Yes and 0 = No)

CT Recommendation (Triage Decision):
"Based on the clinical data provided, would you recommend performing a head CT scan to evaluate for possible intracranial injury in this patient presenting to the emergency department?"
Response: [Answer strictly in binary: 1 = Yes, 0 = No]

Intracranial Injury Prediction (ER Physician Simulation):
"Based on the clinical data provided, and applying your clinical judgment as an emergency department physician, does this patient have a sustained traumatic intracranial injury?"
Response: [Answer strictly in binary: 1 = Yes, 0 = No]

Intracranial Injury Prediction (Rule-Based Model):
"Based on the clinical data provided, and applying a rule-based approach and predefined clinical criteria, does this patient have sustained traumatic intracranial injury?"
Response: [Answer strictly in binary: 1 = Yes, 0 = No]

Here are the binary outputs for the provided case:

CT Recommendation (Triage Decision): 1
Intracranial Injury Prediction (ER Physician Simulation): 0
Intracranial Injury Prediction (Rule-Based Model): 0

Ask anything

ChatGPT can make mistakes. Check important info. See Cookie Preferences.

Supplementary Material S2

Supplementary Material: Univariate and Multi-variable Analysis

Univariate analysis

To identify candidate predictors of intracranial injury in patients with mild traumatic brain injury (mTBI), we conducted univariate analyses for each clinical variable. Categorical variables were analyzed using the Chi-square test, and continuous variables using the independent samples t-test. Variables with a p-value < 0.10 were considered statistically significant and selected for inclusion in the multivariate logistic regression model. The following table displays the results of the univariate analysis:

Variable	Test	p-value
GCS	t-test	0.000045
Age	t-test	0.000300
Amnesia	Chi-square	0.000514
Disorientation	Chi-square	0.001895
Vomiting	Chi-square	0.008702
Headache	Chi-square	0.011704
LOC	Chi-square	0.179413
Alcohol	Chi-square	0.730164
Anticoagulants	Chi-square	0.505356
Antiplatelets	Chi-square	0.532609
Sex	Chi-square	0.608189
Mechanism of injury	Chi-square	0.746616
Seizure	-	n/a
BasilarSigns	-	n/a
Scalp Hematoma / Laceration	Chi-square	0.345678
Cognitive Impairment	-	n/a
Neurological Deficit	-	n/a
Coagulopathy	-	n/a
Prior Neurosurgical intervention	-	n/a

n/a=not applicable due to low number of entries in these categories, LOC=Loss of consciousness

Multivariate logistic regression

Variables with a p-value < 0.10 from the univariate analysis were entered into a binary logistic regression model to assess their independent predictive value for intracranial injury. The dependent variable was the CT result. The results of the logistic regression model are summarized below:

**Variable**	**β Coefficient**	**Standard Error**	**z-value**	**p-value**	**95% CI**
Age	0.0129	0.0111	1.1572	0.2472	-0.0089 to 0.0347
GCS	-2.3007	1.0645	-2.1613	0.0307	-4.3872 to -0.2143
Amnesia	0.9256	0.4847	1.9096	0.0562	-0.0244 to 1.8755
Vomiting	1.0781	0.6040	1.7849	0.0743	-0.1058 to 2.2620
Headache	0.6279	0.4294	1.4624	0.1436	-0.2136 to 1.4694
Disorientation	1.2469	0.8787	1.4191	0.1559	-0.4752 to 2.9691
